# *Helicobacter pylori* infection, atrophic gastritis, and risk of pancreatic cancer: A population-based cohort study in a large Japanese population: the JPHC Study

**DOI:** 10.1038/s41598-019-42365-w

**Published:** 2019-04-15

**Authors:** Mayo Hirabayashi, Manami Inoue, Norie Sawada, Eiko Saito, Sarah K. Abe, Akihisa Hidaka, Motoki Iwasaki, Taiki Yamaji, Taichi Shimazu, Shoichiro Tsugane

**Affiliations:** 1 Department of Global Health Policy, Graduate School of Medicine, The University of Tokyo, 7-3-1 Hongo, Bunkyo-ku, Tokyo 113-0033 Japan; 2 Epidemiology and Prevention Group, Center for Public Health Sciences, National Cancer Center, 5-1-1 Tsukiji, Chuo-ku, Tokyo 104-0045 Japan; 3 Division of Cancer Statistics Integration, Center for Cancer Control and Information Services, National Cancer Center, 5-1-1 Tsukiji, Chuo-ku, Tokyo 104-0045 Japan

## Abstract

*Helicobacter pylori (H. pylori)*, an established risk factor for gastric cancer, is suggested to also play a role in the development of pancreatic cancer; however, the association remains inconclusive. We examined this association among Japanese men and women. *H. pylori* and atrophic gastritis (AG) status were determined serologically, using blood sample collected during health checkups. A total of 20,116 subjects enrolled in the Japan Public Health Center-based Prospective Study Cohort II with available data on *H. pylori* seropositivity (anti-*H. pylori*) and AG were followed until the end of 2010. Cox proportional hazards models were used to calculate the hazard ratios (HR) and 95% confidence intervals (CI), using the information from the baseline survey. During 320,470 person-years of follow-up, 119 cases of pancreatic cancer were identified. No statically significant increase or decrease in pancreatic cancer risk was observed for *H. pylori* and AG status, independently or in combination. In a multivariable-adjusted model, we observed a non-significant decrease in the risk among those who had AG but were anti-*H. pylori* seronegative (HR 0.57, 95% CI 0.31–1.03). In a stratified analysis, we observed a statistically significant increased risk of pancreatic cancer for AG+ (HR 3.64, 95% CI 1.37–9.66), and AG+/anti-*H. pylori*− or AG+/anti-*H. pylori*+ (HR 5.21, 95% CI 1.14–23.87) among current smokers. Non-smokers in all categories of AG and anti-*H. pylori* showed a non-statistical decrease in the risk. There was no statistically significant interaction between *H. pylori* infection, AG status, and smoking status. Our findings suggest *H. pylori* seropositivity and AG, individually or in combination, are not associated with the risk of pancreatic cancer in a general Japanese population. Among current smokers, pancreatic cancer risk increased with AG, regardless of *H. pylori* infection status.

## Introduction

In Japan, pancreatic cancer is the fifth most common cause of cancer death, following lung, stomach, colorectal, and liver^1^. Due to the location of the pancreas, early diagnosis is not easy compared to other digestive tract cancers, which might explain its poor survival rate^2,3^. Pancreatic cancer incidence and death rates increase with age, with a sharp rise after 65^2^.

Age, cigarette smoking and a history of diabetes are the most known risk factors for pancreatic cancer^4^. Although no definite protective factors have been found, intake of fruits and vegetables, and physical exercise are possibly protective^5^. Some studies have shown possible etiological similarities between pancreatic and gastric cancers^6–8^. *Helicobacter pylori* (*H. pylori*) has recently been considered as another possible candidate risk factor for pancreatic cancer. This agent has been declared a group 1 carcinogen by the International Agency for Research on Cancer (IARC), and infection has been found to be strongly associated with the development of gastric-associated diseases, such as peptic ulcer disease and gastric cancer^9^. However, *H. pylori’s* role in the development of pancreatic cancer remains inconclusive^10–13^. One meta-analysis in 2013 including 9 studies showed a 47% increase (summary odds ratio (OR) 1.47, 95% confidence interval (CI) 1.22–1.77) among *H. pylori* infected individuals and pancreatic cancer risk^14^, while a recent meta-analysis including 10 large case-control studies found no significant association^15^. Some previous studies even suggested the possibility of *H. pylori* having a protective effect, particularly infection by cytotoxin-associated gene A (CagA) seropositive *H. pylori* strains, for pancreatic cancer risk^16,17^.

Atrophic gastritis (AG) is a chronic condition characterized by long-term inflammation of the stomach^18,19^. *H. pylori* infection, autoimmune pernicious anaemia, long-term proton pump inhibitor therapy are established etiological risk factors for AG^18^. It has been hypothesised that AG may also be associated with an increased risk of pancreatic cancer through a low-acid production mechanism, leading to bacterial overgrowth, enhancing the promotion of nitroso-compounds^19^. A previous meta-analysis conducted in 2017 could not confirm the association between AG and pancreatic cancer risk but suggested the possibility that the risk may be increasing among the population with AG but are not infected by *H. pylori*^20^.

Given that global ageing trend is likely to continue, investigation of the risk factors for pancreatic cancer is beneficial for better prevention and prediction of the disease. This study aimed to investigate the association between *H. pylori* infection and its related condition, AG, and the pancreatic cancer risk in a Japanese population, using a large-scale prospective study.

## Materials and Methods

### Study population

The study was conducted using the Japan Public Health Center-based Prospective Study (JPHC Study) Cohort II. This cohort was launched in 1993–1994 including with 78,825 Japanese residents (38,740 men and 40,085 women) aged 40–69 years at the beginning of the baseline survey from 6 public health center areas all over Japan^21^. Details of the study design have been described elsewhere^21^. The study protocol was approved by the institutional review board of the National Cancer Center, Japan (Approval Number: 2001–021) and The University of Tokyo (approval number: 10508). All methods used in this study were performed by the relevant guidelines and regulations.

### Baseline survey

**Figure 1:**
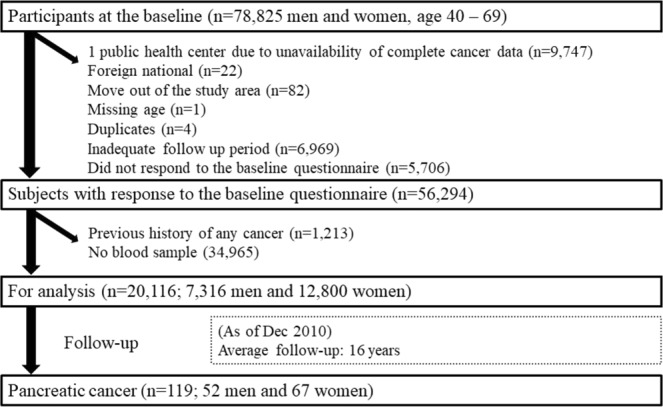
Study participant selection process.

A self-administered questionnaire regarding lifestyle factors was completed at the baseline of the cohort. The participants were informed of the objectives of the study, and those who completed the survey questionnaire were regarded as consenting to participate in the study. Figure 1 shows the study particpants selection process. Of 78,825 participants at the baseline, participants from one public health center area (Suita, n = 9,747) were excluded due to the unavailability of complete cancer data. We excluded foreign nationals (n = 22), move out of the study area before the study starting point (n = 82), missing age (n = 1), duplicates (n = 4), or those with inadequate follow-up data (n = 81). We further excluded individuals who had died, moved out of the study area, or had an unknown date of diagnosis before the starting point (n = 6,969), and non-respondents to the baseline questionnaire (n = 5,706). Among those who responded to the baseline questionnaire, 38% (n = 21,329) voluntarily provided 10 mL of blood during health checkups provided by their local government. Samples were divided into four tubes for plasma and buffy layer and stored at −80 °C until analysis. Subjects who reported a history of any cancer (n = 1,213) were excluded from the study, leaving 20,116 individuals (7,316 men and 12,800 women) for the analysis.

### Laboratory analysis

*H. pylori* infection and AG were defined using biomarkers *H. pylori* seropositivity (anti-*H*. pylori) and pepsinogen (PG) I and II respectively. Levels of Immunoglobin G (IgG) were measured using an enzyme immunoassay (E Plate “Eiken” *H. pylori* Antibody II; Eiken Kagaku, Tokyo, Japan)^22^. An IgG titer of anti-*H. pylori* ≥10 U/mL was considered *H. pylori* seropositivy^22^. The latex agglutination technique was used to determine the plasma levels of PG I and II (LZ test “Eiken” Pepsinogen I, II; Eiken Kagaku, Tokyo, Japan)^22^. A subject was defined as AG-positive if PG I ≤70 ng/mL, and PG I/II ratio ≤3.0^22^. Miki conducted a meta-analysis in 2006^23^ for the sensitivity and specificity of the serum pepsinogen test method using results from 42 studies. The combined sensitivity and false-positive rates for PGI ≤70 ng/mL and PG I:II ratio ≤3.0 were 77% and 27%, respectively^23^. The positive predicted value ranged from 0.77% to 1.25%, while the negative predictive value varied from 99.08% and 99.90%^23^. CagA seropositivity was not examined due to limited availability of stored blood samples. Using these criteria, the study subjects were further divided into three groups according to a combination of AG status and anti-*H. pylori*; AG-negative and *H. pylori* seronegative (AG−/ anti-*H. pylori*−); AG-negative and *H. pylori* seropositive (AG−/anti-*H. pylori*+); AG-positive and *H. pylori* seropositive (AG+/anti-*H. pylori*+).

### Follow-up and identification of pancreatic cancer cases

Subjects were followed from the date of the baseline survey until December 31^st^, 2010. Residence and survival status of the subjects was confirmed through the residential registry. The incidence of pancreatic cancer was identified through active patient notification from major local hospitals in each study area and linking with population-based cancer registries. Death certificates were used to supplement the information on cancer incidence. Cases of pancreatic cancer were classified using the International Classification of Diseases for Oncology, 3^rd^ edition, code C25^24^.

### Statistical analysis

Study participants were censored on the date of pancreatic cancer diagnosis, move-out from the study area, death, or December 31^st^, 2010, whichever came first. Subjects’ characteristics at the baseline were compared independently by anti-*H. pylori* and AG status, and also by a combination of the two biomarkers. Differences in baseline characteristics between anti-*H. pylori* and AG status were analyzed using analysis of variance or *χ*^2^-test. Cox proportional hazards regression models were used to estimate the hazard ratios (HRs) and their 95% CI using attained age as the time scale due to the strong association between pancreatic cancer risk and age. Participants who tested negative to the biomarkers at the baseline of the study were used as the reference group. Covariates included were based in associations found in previous studies^25^. Model 1 adjusted for public health center areas (six areas treated as strata) and gender, while model 2 further adjusted for body-mass index (BMI) calculated using measured height and weight (<25.0 kg/m^2^, ≥25.0–<27.0 kg/m^2^, ≥27.0 kg/m^2^), self-reported history of diabetes (yes, no), self-reported physical activity (continuous, metabolic equivalent of task (METs)), self-reported alcohol consumption (never and formal, occasional, <150 g/week, ≥150 g/week), self-reported family history of pancreatic cancer (yes, no), and self-reported smoking status (never, former, current), in addition to the confounders in model 1. We conducted individual stratified analyses for each pancreatic cancer risk factors to see how *H. pylori* seropositivity and AG status affected the pancreatic cancer risk differently according to lifestyle habits. Interactions were considered between *H. pylori* infection, AG status and each of the covariates included in the analysis by running a regression model with an interaction term, then conducting a Wald test for interaction. All analyses were conducted using Stata version 13.0 (StataCorp LP).

## Results

During 320,470 person-years of follow-up (mean 16 years), 119 cases (52 men and 67 women) of newly diagnosed pancreatic cancer were identified among 20,116 subjects. Table 1 shows the baseline characteristics of cohort participants by *H. pylori* seropositivity and AG categories. We combined never-and past- alcohol drinkers due to a small number of participants in past drinker category (n = 383, 1.9% of the total participants). Men represented 36% of the total participants. AG−/anti-*H. pylori*+ category had the highest percentage of men (39.1%),  habitual drinkers (≥150 g of ethanol per week, 15.5%), and current smokers (18.3%). In all categories, the majority of the subjects were within normal BMI range (<25 kg/m^2^). At the baseline, 13,752 subjects (68%) and 8,470 (42%) were found to be anti-*H. pylori* seropositive and AG positive, respectively. Mean PGI value for our study was 53.4 ± 29.0 ng/mL; minimum PGI value observed was 2 ng/mL and the maximum PGI value was 606.4 ng/mL.

**Table 1:** Baseline characteristics of study participants by *H. pylori* infection and AG status, JPHC Cohort II (1993–2010)

	Total participants	AG−/anti-*H. pylori*−	AG−/anti-*H. pylori*+	AG+/anti-*H. pylori*+ or AG+/anti-*H. pylori−*^*a*^	P*
Subjects	20,116	5,716	5,930	8,470	
Total person time (person-years)	320,469.83	91,563.88	95,107.09	133,798.86	
Age (y, mean ± SD)	56.9 ± 8.2	55.7 ± 8.5	56.1 ± 8.2	58.2 ± 7.9	<0.001
Gender, men (%)	7,316 (36.4)	1,859 (32.5)	2,316 (39.1)	3,141 (37.1)	<0.001
Smoking status (%)					
Never	14,066 (69.9)	4,181 (73.1)	4,072 (68.7)	5,813 (68.6)	0.14
Former	2,356 (11.7)	531 (9.3)	744 (12.5)	1,081 (12.8)	
Current	3,591 (17.8)	976 (17.1)	1,085 (18.3)	1,530 (18.1)	
Missing information	103 (0.5)	28 (0.5)	29 (0.5)	46 (0.5)	
Body-mass index (BMI) (kg/m^2^) (%)					
<25	13,956 (69.4)	3,835 (67.1)	4,078 (68.8)	6,043 (71.3)	0.001
≥25–<27	3,295 (16.4)	972 (17.0)	986 (16.6)	1,337 (15.8)	
≥27	2,856 (14.2)	906 (15.8)	864 (14.6)	1,086 (12.8)	
Missing information	9 (0.04)	3 (0.05)	2 (0.03)	4 (0.05)	
Alcohol consumption (%)					
Never	11,949 (59.4)	3,440 (60.2)	3,416 (57.6)	5,093 (60.1)	<0.001
Past	383 (1.9)	94 (1.6)	118 (2.0)	171 (2.0)	
Occasional	1,235 (6.1)	390 (6.8)	370 (6.2)	475 (5.6)	
<150 g/week	3,023 (15.0)	824 (14.4)	950 (16.0)	1,249 (14.7)	
≥150 g/week	3,032 (15.1)	818 (14.3)	918 (15.5)	1,296 (15.3)	
Missing information	494 (2.5)	150 (2.6)	158 (2.7)	186 (2.2)	
History of diabetes mellitus type II (%)					
Yes	1,043 (5.2)	323 (5.6)	333 (5.6)	387 (4.6)	<0.001
No	19,073 (94.8)	5,393 (94.3)	5,597 (94.4)	8,083 (95.4)	
Pancreatic cancer cases	119	30	22	67	

AG: Atrophic gastritis anti-*H. pylori*: *H. pylori* seropositivity AG+: PG I level ≤70 ng/mL combined with a ratio of PGI and II ≤3.0 *H. pylori*+: Level of serum IgG ≥10 U/mL.

^a^This group included subjects with AG+/anti-*H. pylori*− and AG+/anti-*H. pylori*+.

*χ^2^ Test for ordinal qualitative variables and linear regression for continuous variables.

Table 2 shows the HR and 95% CI for anti-*H. pylori* and AG status and the risk of pancreatic cancer, using attained age as a time scale. Those who tested negative to each agent were used as a reference. AG is considered the endpoint of chronic gastritis caused by *H. pylori* infection^26^; therefore, AG+/anti-*H. pylori*− category was combined with AG+/anti-*H. pylori*+ category.

**Table 2:** Association between *H. pylori* infection status and risk of pancreatic cancer, JPHC Cohort II (1993–2010)

	Subjects	Person-time (person-years)	Cases	Model 1 HR (95% CI)^a^	Model 2 HR (95% CI)^b^
Total participants	20,116	320,469.83	119		
AG−	11,646	186,670.97	52	1 (ref)	1 (ref)
AG+	8,470	133,798.86	67	1.22 (0.81–1.85)	1.24 (0.82–1.88)
anti-*H. pylori*−	6,364	101,305.12	36	1 (ref)	1 (ref)
anti-*H. pylori*+	13,752	219,164.71	83	0.76(0.49–1.17)	0.76 (0.49–1.18)
AG and anti-*H. pylori* combination					
AG−/anti-*H. pylori*−	5,716	91,563.88	30	1 (ref)	1 (ref)
AG−/anti-*H. pylori*+	5,930	95,107.09	22	0.55 (0.31–1.01)	0.57 (0.31–1.03)
AG+/anti-*H. pylori*− or AG+/anti-*H. pylori*+ ^c^	8,470	133,798.86	67	0.93 (0.58–1.49)	0.97 (0.59–1.54)

Cox proportional hazards model was used. ^a^Model1 included public health center area and gender. ^b^Model 2 included metabolic equivalent of task (METs); alcohol consumption; smoking status; family history of pancreatic cancer; body-mass index (BMI) category; history of diabetes mellitus type II, in addition to adjustments used in model 1.

^c^This group included subjects with AG+/anti-*H. pylori*− and AG+/anti-*H. pylori*+ AG: Atrophic gastritis anti-*H. pylori*: *H. pylori* seropositivity AG+: PGI level ≤70 ng/mL combined with a ratio of PG I and II ≤3.0\anti-*H. pylori*+*:* Level of serum IgG ≥10 U/mL.

No statistical association was found between AG and anti-*H. pylori* status and pancreatic cancer risk, even when the model was adjusted for potential confounders. A borderline decrease in risk was observed among AG−/*H. pylori*+ subjects (HR 0.57, 95% CI 0.31–1.03).

Table 3 shows the association between *H. pylori* infection and the risk of pancreatic cancer by individually stratifying for smoking status, alcohol consumption, BMI, and history of diabetes. Among current smokers, we saw a statistically significant increase in the risk of pancreatic cancer for AG+ (HR 3.64, 95% CI 1.37–9.66) and AG+/anti-*H. pylori*− or AG+/anti-*H*. pylori+ subjects (HR 5.21, 95% CI 1.14–23.87). There were no statistically significant interactions between *H. pylori* infection, AG status, smoking status, alcohol consumption, BMI and history of diabetes.

**Table 3:** Stratified analysis by risk factors for an association between *H. pylori* infection and pancreatic cancer risk according to AG and *H. pylori* infection status, JPHC Cohort II (1993–2010)

	Subjects (n = 20,116)	Person time (person years) (320,469.83)	AG− (n = 11,646)		AG+ (n = 8,470)		*anti-H. pylori*− (n = 6,364)		*anti-H. pylori*+ (n = 13,752)		AG and anti-*H. pylori* status					
											AG−/anti-*H. pylori*− (n = 5,716)		AG−/anti-*H. pylori*+ (n = 5,930)		AG+/anti-*H. pylori*− *or AG*+*/anti-H. pylori*+^a^ (n=8,470)	
			Cases	HR (95% CI)	Cases	HR (95% CI)	Cases	HR (95% CI)	Cases	HR (95% CI)	Cases	HR (95% CI)	Cases	HR (95% CI)	Cases	HR (95% CI)
Smoking status																
Never	14,066	229,280.80	37	1 (ref)	36	0.90 (0.53–1.53)	23	1 (ref)	50	0.66 (0.39–1.12)	22	1 (ref)	15	0.52 (0.25–1.05)	36	0.68 (0.38–1.22)
Past	2,356	36,050.32	6	1 (ref)	10	1.38 (0.41–4.72)	5	1 (ref)	11	0.85 (0.22–3.24)	4	1 (ref)	2	0.43 (0.07–2.60)	10	0.91 (0.22–3.75)
Current	3,591	53,520.19	8	1 (ref)	20	3.64 (1.37–9.66)	7	1 (ref)	21	1.41 (0.50–4.02)	3	1 (ref)	5	1.81 (0.32–10.36)	20	5.21 (1.14–23.87)
Body-mass index (BMI) (kg/m)																
<25	13,956	220,752.70	38	1 (ref)	50	1.24 (0.76–2.00)	26	1 (ref)	62	0.82 (0.49–1.36)	21	1 (ref)	17	0.67 (0.33–1.29)	50	1.01 (0.57–1.77)
≥ 25–<27	3,295	53,252.26	5	1 (ref)	7	0.70 (0.17–2.90)	4	1 (ref)	8	0.35 (0.08–1.51)	4	1 (ref)	1	0.15 (0.01–1.53)	7	0.32 (0.06–1.54)
≥30	2,856	46,327.27	9	1 (ref)	10	1.39 (0.45–4.29)	6	1 (ref)	13	0.62 (0.19–2.04)	5	1 (ref)	4	0.45 (0.08–2.57)	10	0.99 (0.27–3.57)
Ethanol consumption																
Never and past	11,949	198,534.54	31	1 (ref)	37	0.92 (0.16–5.37)	17	1 (ref)	51	0.91 (0.51–1.64)	16	1 (ref)	15	0.82 (0.39–1.72)	37	0.85 (0.45–1.63)
Occasional	1,235	20,125.81	4	1 (ref)	3	1.71 (0.22–13.46)	4	1 (ref)	3	0.12 (0.01–1.55)	3	1 (ref)	1		3	0.58 (0.06–5.55)
<150 g/week	3,023	47,397.47	8	1 (ref)	10	1.83 (0.61–5.49)	6	1 (ref)	12	0.97 (0.29–3.20)	5	1 (ref)	3	0.38 (0.07–2.13)	10	1.17 (0.33–4.13)
≥150 g/week	3,032	46,651.33	7	1 (ref)	17	2.12 (0.83–5.43)	7	1 (ref)	17	0.73 (0.27–1.95)	4	1 (ref)	3	0.51 (0.11–2.31)	17	1.49 (0.47–4.72)
History of diabetes mellitus type 2																
No	19,073	304,988.96	44	1 (ref)	61	1.32 (0.85–2.04)	32	1 (ref)	73	0.76 (0.48–1.20)	26	1 (ref)	18	0.54 (0.28–1.02)	61	0.99 (0.60–1.64)
Yes	1,043	15,490.87	8	1 (ref)	6	0.63 (0.14–2.86)	4	1 (ref)	10	1.39 (0.26–7.44)	4	1 (ref)	4	1.44 (0.24–8.67)	6	0.74 (0.13–4.24)

Cox proportional hazards model was used. Model included public health center area, gender, metabolic equivalent of task (METs); alcohol consumption; smoking status; family history of pancreatic cancer; body-mass index (BMI) category; history of diabetes mellitus type II anti-*H. pylori*: *H. pylori* seropositivity AG+: PGI level ≤70 ng/mL combined with a ratio of PG I and II ≤3.0 anti-*H. pylori*+: Level of serum IgG ≥10 U/mL.

^a^This group included subjects with AG+ /anti-*H. pylori-* and AG+ /anti-*H. pylori*+.

## Discussion

To best of our knowledge, this is the first prospective cohort study to investigate *H. pylori* infection status and the risk of pancreatic cancer incidence in a Japanese population. When stratified by smoking status, the risk of pancreatic cancer among current smokers with AG statistically increased, regardless of *H. pylori* infection status.

We observed a non-statistically significant decrease in the risk of pancreatic cancer for AG−/anti-*H. pylori*+ subjects. Although the exact mechanism of how *H. pylori* seropositivity lowers pancreatic cancer risk is unclear, one hypothesis proposed suggests suppression of appetite by *H. pylori* infection leading to a reduction of ghrelin, ultimately lowering body weight, reducing cases of pancreatic cancer caused by obesity^27^.

Another possibility is the infection by CagA seropositive *H. pylori* strains may be working as a protective factor for pancreatic cancer development. Risch *et al*.^16^ showed a statistically significant risk decrease for pancreatic cancer among *H. pylori* and CagA seropositive in a Chinese population (OR 0.66, 95% CI 0.53–0.81). A meta-analysis conducted based on 2,049 cases and 2,861 controls also showed a reduction in pancreatic cancer risk among those who are infected with *H. pylori* (summary OR 0.62, 95% CI 0.49–0.76) and CagA seropositive (summary OR 0.66, 95% CI 0.52–0.80) in Asian population^28^. Evidence from mouse models have suggested SHP-2, a tyrosine phosphate expressed in most embryonic and adult tissues and an intracellular target of *H. pylori* CagA protein, may be regulating glucose and lipid metabolism by suppressing insulin signalling in hepatocytes^29^, suppressing tumour proliferation in the pancreas. Eradication of CagA seronegative *H. pylori* among patients with duodenal ulcer returns their hyperchlorhydria to normal^30,31^; in contrast, eradication of CagA seropositive *H. pylori* among corpus AG patients returns the stomach environment to hypo- or achlorhydria to normal^32^. Since gastric acidity promotes secretion of bicarbonate and fluid from pancreatic ductal cells^33^, it allows CagA seronegative *H. pylori* to survive in pancreatic ductular epithelium^16^. An animal model has shown that excess production of pancreatic bicarbonate and fluid increased the ductular cell dysplasia and adenocarcinoma^34^. This suggests the possibility of difference in gastritis acidity caused by *H. pylori* stains may affect in the pancreatic cancer risk^16^. However, because we could not measure CagA seropositivity due to limited availability of stored blood samples, we were not able to test the hypothesis.

Stratified analysis by established pancreatic cancer risk factors showed a statistically significant increase in the pancreatic cancer risk among current smokers with AG. This result is inconsistent with a previous Finnish cohort study that recruited current male smokers to look at the association between AG and pancreatic cancer and found no association, whether AG was diagnosed serologically or histologically^19^. Serum PG levels are known to be affected depending on demographic characteristics such as gender, age, smoking, alcohol consumption, and dietary habits^35^, leading to various cut-off values of serum PG depending on populations. In our study, we used the threshold for defining a high-risk population in Japan^36^ (PGI ≤ 70 ng/mL and a ratio of PGI and PGII ≤ 3), proposed by Miki *et al*.^23,37,38^, to define AG. In European nations, the often used serum PGI cut-off value is ≤25 ng/mL, with a ratio of PGI and PGII < 3^39^. It is possible that the differences in the findings between countries are due to the difference in PGI cut-offs used to define AG.

No epidemiological evidence has been found to clarify how AG is associated with pancreatic cancer risk. Truan *et al*.^40^ found pepsinogen expression in 38% pancreatic cancer cases, while another study found that gastrin, a gastrointestinal peptide, had a proliferative effect on pancreatic cancer cells^41^. AG is often caused by *H. pylori* infection^42–44^. The amount of *H. pylori* present in the stomach reduces as intestinal metaplasia develop, spreading in the presence of chronic AG^45–47^, resulting in a negative result in the antibody test^48^. IgG anti-*H. pylori* seronegative status among those who are AG positive implies prior *H. pylori* infection, since *H. pylori* cannot survive in the atrophic or intestinal metaplasia mucosa^49^. Previous studies have shown those with advanced gastritis with loss of *H. pylori* are at a higher risk of developing gastric cancer^48,49^. This may be the reason why the increased risk for pancreatic cancer was observed among AG positive but *H. pylori* negative individuals in a previous study^20^. AG therefore may have a causative effect on pancreatic cancer development, through the extra-gastric or systemic effect of *H. pylori*^19^.

A major strength of this study is its prospective cohort design, with subjects recruited from a large sample of the general population. The high response rate and low loss to follow-up increased the generalizability of the conclusions and reduced selection bias. We used the incidence of pancreatic cancer as an endpoint rather than death since it directly measured the risk of pancreatic cancer. Finally, because the *H. pylori* infection rate in the Japanese population is high for those born prior to 1950 (over 70%)^50^, the country provides a suitable setting for examining the association of *H. pylori*, AG, and pancreatic cancer.

There are several limitations. The cases of pancreatic cancer found during the follow up period were relatively small (n = 119). However, we believe that with an average of 16 years of follow-up, sufficient numbers of pancreatic cancer cases were identified. Furthermore, because the development of pancreatic cancer is a rare event (Age-standardized rate per 100,000: 9.7)^51^, the incidence observed during this study period is believed to be acceptable. We also had to exclude 34,965 subjects from the analysis since they did not provide a blood sample, and therefore their *H. pylori* and AG status could not be observed. Men comprised 36% of the study population, which may have led to a gender-biased result. *H. pylori* infection status was defined using serum antibody-based tests, which are relatively inexpensive, rapidly performed, and cause minimal discomfort to the subjects^52^. However, one of the limitations of using serology test is its inability to differentiate between past and present *H. pylori * infections^53^. Although *H. pylori* infection is said to be associated with low socioeconomic status (SES)^54^, due to the unavailability of the data, we were not able to include SES into our analysis. This study also did not consider blood group antigens, a potential risk factor for pancreatic cancer incidence reported by several studies including a study conducted by Wolpin *et al*.^55^, which reported compared to those with blood type O, blood type A and B have a significantly increased risk for pancreatic cancer. Finally, the association may have been confounded by additional unmeasured or unknown risk factors.

In summary, *H. pylori* infection and AG was not associated with the risk of pancreatic cancer in a general Japanese population, individually or in combination; however, among current smokers with AG, a significant increase in the risk of pancreatic cancer was observed. Further investigation in larger cohorts, especially in Asian countries, will provide a more comprehensive evaluation.
