# Author Correction: *Helicobacter pylori* infection, atrophic gastritis, and risk of pancreatic cancer: A population-based cohort study in a large Japanese population: the JPHC Study

**DOI:** 10.1038/s41598-020-69880-5

**Published:** 2020-07-28

**Authors:** Mayo Hirabayashi, Manami Inoue, Norie Sawada, Eiko Saito, Sarah K. Abe, Akihisa Hidaka, Motoki Iwasaki, Taiki Yamaji, Taichi Shimazu, Shoichiro Tsugane

**Affiliations:** 1 Department of Global Health Policy, Graduate School of Medicine, The University of Tokyo, 7-3-1 Hongo, Bunkyo-ku, Tokyo, 113-0033 Japan; 2 Epidemiology and Prevention Group, Center for Public Health Sciences, National Cancer Center, 5-1-1 Tsukiji, Chuo-ku, Tokyo, 104-0045 Japan; 3 Division of Cancer Statistics Integration, Center for Cancer Control and Information Services, National Cancer Center, 5-1-1 Tsukiji, Chuo-ku, Tokyo, 104-0045 Japan

Correction to: *Scientific Reports* 10.1038/s41598-019-42365-w, published online 15 April 2019

This Article contains errors in Table 3.

The subheading of the row for Body-mass index is incorrectly written as ‘Body-mass index (BMI) (kg/m)’.

The correct row subheading should read ‘Body-mass index (BMI) (kg/m^2^)’.

Additionally, the third subheading of the ‘Body-mass index (BMI) (kg/m^2^)’ section is incorrectly given as ‘≥ 30’.

The correct row subheading should read ‘≥ 27’.

